# Treating Chronic Iliac Vein Stent Occlusion in an Office-Based Lab Setting

**DOI:** 10.7759/cureus.61298

**Published:** 2024-05-29

**Authors:** Harneet S Sangha, Ryan Nolan, Edward J Arous

**Affiliations:** 1 Department of Vascular Surgery, The Vascular Care Group, Worcester, USA

**Keywords:** office-based lab, surgery, post thrombotic syndrome, recanalization, iliac vein

## Abstract

Iliac vein stenting is performed when sufficient venous patency is not achieved via angioplasty or lysis. Iliac vein stenting is known to be effective; however, occlusion of the stent occurs occasionally. There is a lack of effective treatment options for those with failed prior venous stents, and traditional methods may involve the removal of the stent and surgical reconstruction. We present a patient with a right leg post-thrombotic syndrome and narcotic abuse after occlusion of a previously placed right common iliac/external iliac vein stent 25 years prior. After transfer to an office-based lab (OBL), femoral vein access was achieved. Then, a second stent was deployed adjacent to the previously chronically thrombosed stent. Imaging confirmed adequate deployment of the new stent and venous flow. Treatment resulted in a significant decrease in patient pain and cessation of narcotics. We demonstrate successful recanalization of a right iliac vein thrombosis via parallel deployment of a stent adjacent to a chronically thrombosed stent.

## Introduction

As healthcare expenses in the United States keep rising, there's been a shift of procedures from hospitals to lower-cost outpatient or office-based settings. To address cost concerns, many physicians have established office-based laboratories (OBLs) to conduct low and moderate-risk procedures. Additionally, over the past decade, there has been a notable rise in the quantity of these OBLs, accompanied by a migration of traditional inpatient procedures to outpatient settings. This transition has led to reduced costs, heightened patient satisfaction, and improved efficiency [1]. In particular, arterial and venous interventions have seen a significant shift towards outpatient settings [2]. 

Iliac vein stenting is performed when angioplasty and/or lysis alone fail to achieve sufficient venous patency in addressing three prevalent conditions: nonthrombotic iliac vein lesions (NIVLs), such as compression (commonly referred to as May-Thurner syndrome); thrombotic occlusions, or outflow obstructions in individuals experiencing symptomatic reflux associated with chronic venous insufficiency (CVI) [3]. However, one complication of iliac vein stenting is stent thrombosis. As reported in a recent study by Jayaraj et al., occlusion can be acute (<30 days; 31%) or chronic (>30 days; 69%) [4]. The traditional treatment for stent occlusion necessitates wire recanalization followed by balloon angioplasty and addressing the underlying stent thrombosis, including potential extension of the stent proximally and/or distally [4]. 

Failure to treat occluded venous stents can lead to post-thrombotic syndrome (PTS), where patients typically exhibit symptoms including edema, chronic leg pain, swelling, skin alterations, and a sensation of heaviness in the limb previously affected by deep vein thrombosis (DVT) [5]. These symptoms stem from compromised venous blood flow due to damaged venous valves and obstructive conditions resulting from a prior DVT episode as well as occlusion of previous venous stents. Additionally, diagnosing and recognizing PTS can be difficult because symptoms vary from person to person. Typically, patients may experience pain, swelling, edema, heaviness, and changes in the skin of the affected limb. These symptoms often improve when resting or elevating the limb but worsen when standing or walking. Other signs to watch for include telangiectasia, hyperpigmentation, eczema, varicose veins, ulcers, or lipodermatosclerosis [6]. 

We demonstrate successful recanalization of a right iliac vein thrombosis via parallel deployment of a stent adjacent to a chronically thrombosed stent. Additionally, we discuss the safety of venous stenting in the OBL setting.

## Case presentation

**Figure 1:**
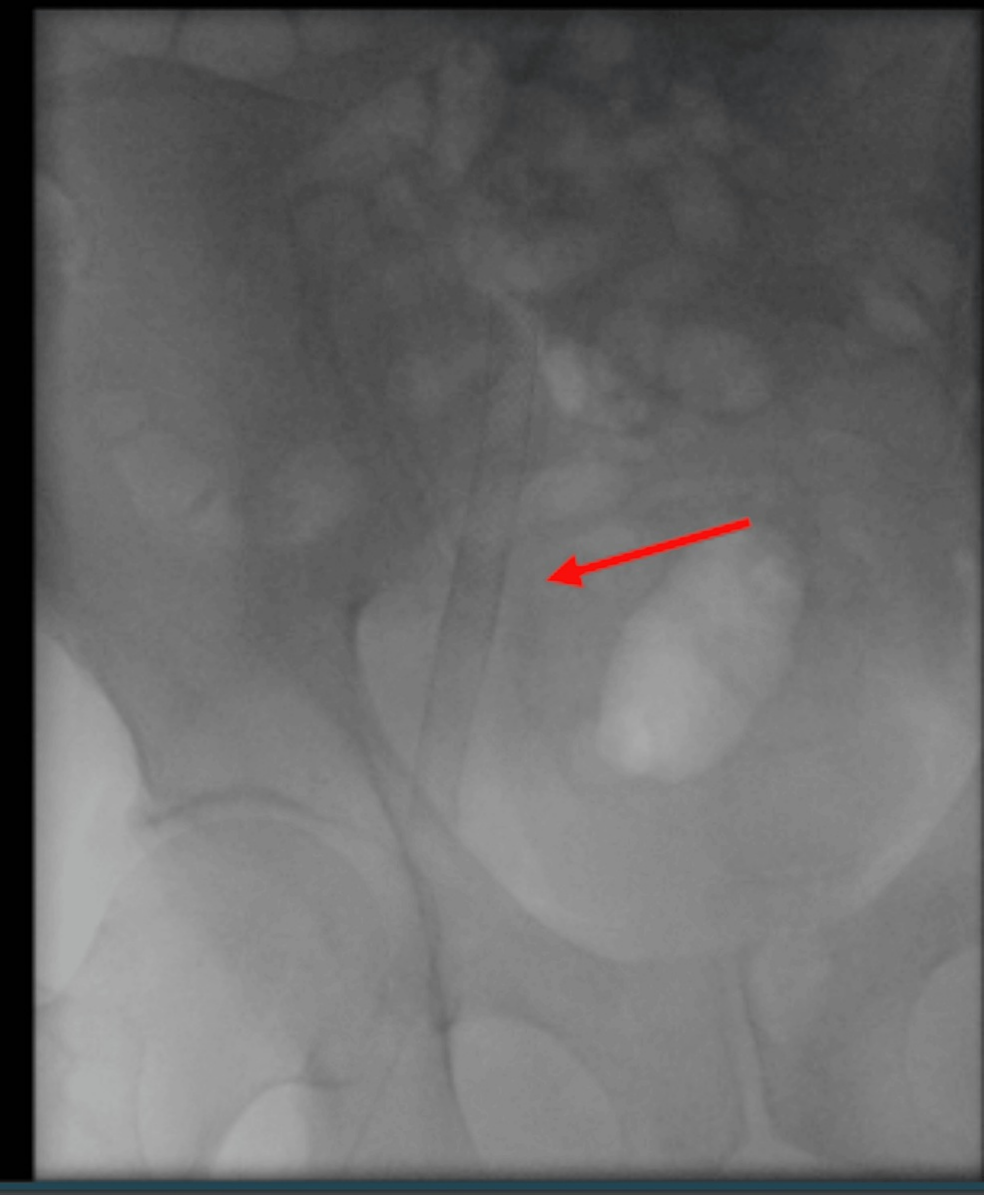
Initial venogram demonstrating previous right common iliac/external iliac Wallstent

**Figure 2:**
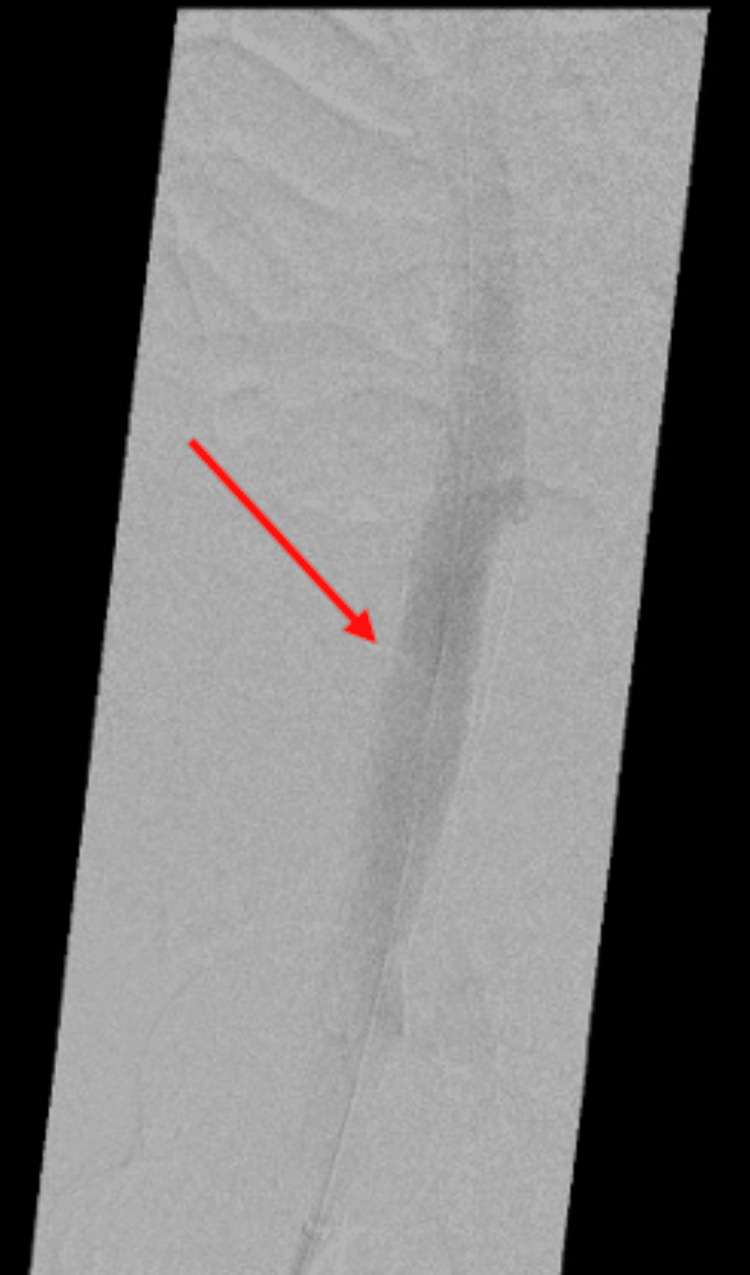
Initial venogram with drainage with collaterals through the right internal iliac vein

**Figure 3:**
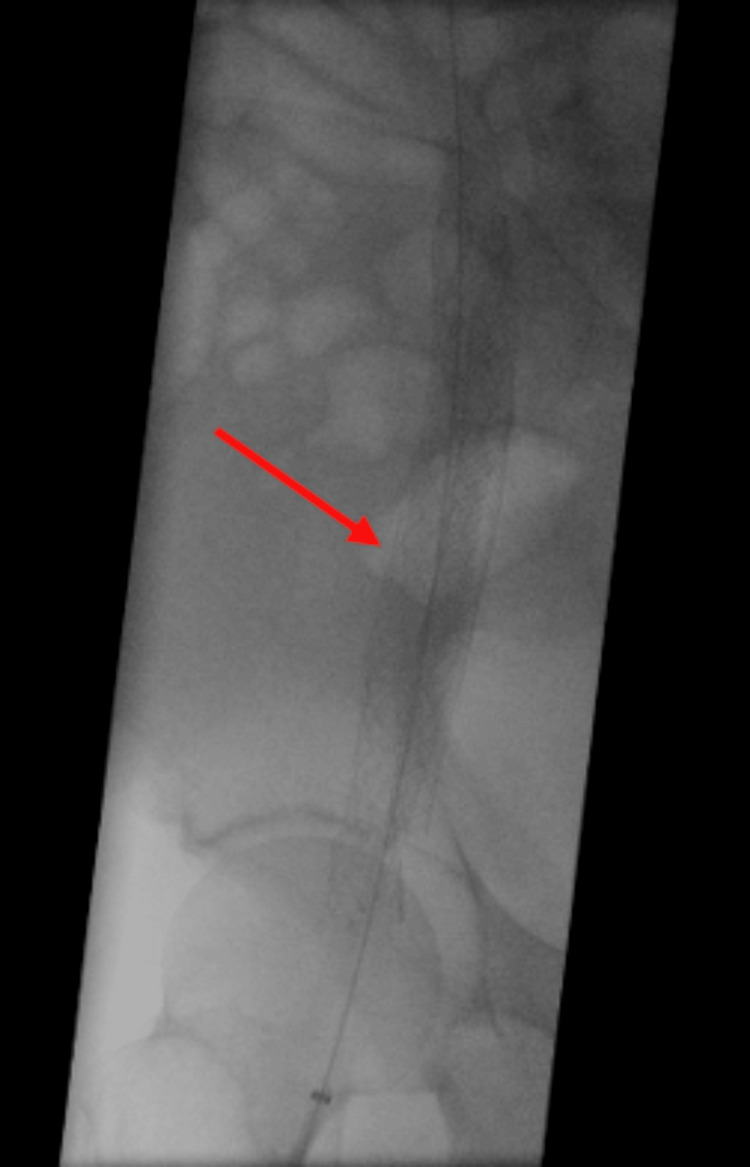
Completion venogram with 16mm Abre stent deployed proximal and distal to previous Wallstent

​​A 46-year-old man with a history of right iliac vein stenting in 1998 with a 10mm Wallstent (Boston Scientific, Marlborough, Massachusetts) presented with chronic right leg post-thrombotic syndrome and narcotic abuse (Figure 1). His previous right common iliac/external iliac vein stent had occluded shortly after initial placement but had not recanalized (Figure 2). He was taken to the OBL, and femoral venous access was obtained. Catheter and wire manipulation were used to navigate across the right iliac vein occlusion. Intravascular ultrasonography confirmed wire transit adjacent to the chronically occluded stent. A 16mm Abre stent (Medtronic, Dublin, Ireland) was deployed from the right common iliac to the right common femoral vein with subsequent venography and Intravascular ultrasound, demonstrating spontaneous venous flow and complete stent expansion (Figure 3). The patient reported an 80% reduction in pain 40 days after stenting and discontinued his narcotic regimen 30 days after stenting. The patient remains on therapeutic anticoagulation, and the iliac vein stent remains patent on surveillance duplex 15 months after deployment.

## Discussion

The popularity of OBLs has been on the rise between 2013 and 2015. Approximately 5298 healthcare professionals across the country conducted endovascular arterial and venous stenting procedures during this timeline. Among them, 20.8% exclusively conducted these procedures in an OBL setting [7]. While there's a scarcity of literature specifically addressing the safety of stenting in OBLs, a few studies have explored this question. In a 2019 analysis encompassing 24 studies on iliocaval venous stenting, Williams and Dillavou noted a 9.5% complication rate overall [8]. In another recent study, the authors observed major complications, defined as those requiring hospitalization, such as allergic reactions, one episode each of atrial fibrillation, supraventricular tachycardia, and chest pain, as well as one case of acute stent occlusion. These complications occurred in 0.41% of the patients. [9]. This rate suggests that iliac vein stenting in the OBL setting is safe.

Chronic venous occlusions frequently result from deep vein thrombosis (DVT) in the iliofemoral venous distribution. In contrast to the femoral, popliteal veins, which often recanalize at high rates with conservative anticoagulation therapy alone, the iliofemoral veins exhibit unacceptably low recanalization rates, leading to narrow, irregular lumens [10]. Chronic iliac vein stent occlusions lead to more severe symptoms compared to obstructions in more distal segments. This includes ambulatory venous hypertension, the emergence of incompetent valves, venous claudication, and often tissue loss [11]. Historical treatment for chronic blockages in the iliofemoral region involved surgery. However, this is considered highly invasive, with uncertain long-term effectiveness, and often requiring lifelong anticoagulant therapy. As a result, endovascular treatment with stenting is now considered the mainstay for chronic iliofemoral obstruction. The appeal lies in the invasive nature and the high rates of success in maintaining vessel patency. However, there is limited literature on the treatment for stent occlusion following venous intervention. 

Several techniques have been used to treat occluded stents, such as using basic guidewire and catheter techniques to mechanically recanalize stents. In recent years, numerous advanced endovascular devices have been created for treating chronic arterial blockages, employing methods like blunt microdissection, radiofrequency, and laser; however, their use has not been published widely in the literature [8-11]. We herein demonstrate an alternative method for treating chronic right iliac vein thrombosis due to an occluded stent via parallel deployment of a stent adjacent to a chronically thrombosed stent. Parallel stent placement offers a superior method for treating occluded stents by providing a new conduit for blood flow without the need to remove the existing, thrombosed stent, thereby minimizing the risk of vessel injury and complications. Additionally, this technique can be performed with greater ease and precision, reducing procedure time and improving patient outcomes compared to more invasive or complex recanalization methods.

## Conclusions

Our case report highlights the successful recanalization of a right iliac vein thrombosis in a patient with post-thrombotic syndrome and narcotic abuse through the innovative approach of deploying a second stent adjacent to a chronically occluded stent. This procedure, conducted in an OBL, not only effectively alleviated the patient's symptoms but also eliminated the need for traditional, more invasive treatments. Moreover, our experience contributes to the growing body of evidence supporting the safety and efficacy of venous interventions in OBL settings. As the healthcare landscape continues to evolve towards outpatient care, such minimally invasive techniques offer promising solutions for complex venous pathologies.
